# Immunotherapy and Targeting the Tumor Microenvironment: Current Place and New Insights in Primary Pulmonary NUT Carcinoma

**DOI:** 10.3389/fonc.2021.690115

**Published:** 2021-09-30

**Authors:** Xiang Li, Hui Shi, Wei Zhang, Chong Bai, Miaoxia He, Na Ta, Haidong Huang, Yunye Ning, Chen Fang, Hao Qin, Yuchao Dong

**Affiliations:** ^1^ Department of Respiratory and Critical Care Medicine, Changhai Hospital (The First Affiliated Hospital of Naval Medical University), Naval Medical University (Second Military Medical University), Shanghai, China; ^2^ Department of Pathology, Changhai Hospital (The First Affiliated Hospital of Naval Medical University), Naval Medical University (Second Military Medical University), Shanghai, China

**Keywords:** nuclear protein of testis carcinoma, NUT carcinoma, midline carcinoma, pulmonary, tumor microenvironment, immunotherapy, immune checkpoint inhibitor

## Abstract

Primary pulmonary nuclear protein of testis carcinoma is a rare and highly aggressive malignant tumor. It accounts for approximately 0.22% of primary thoracic tumors and is little known, so it is often misdiagnosed as pulmonary squamous cell carcinoma. No effective treatment has been formed yet, and the prognosis is extremely poor. This review aims to summarize the etiology, pathogenesis, diagnosis, treatment, and prognosis of primary pulmonary nuclear protein of testis carcinoma in order to better recognize it and discuss the current and innovative strategies to overcome it. With the increasing importance of cancer immunotherapy and tumor microenvironment, the review also discusses whether immunotherapy and targeting the tumor microenvironment can improve the prognosis of primary pulmonary nuclear protein of testis carcinoma and possible treatment strategies. We reviewed and summarized the clinicopathological features of all patients with primary pulmonary nuclear protein of testis carcinoma who received immunotherapy, including initial misdiagnosis, disease stage, immunohistochemical markers related to tumor neovascularization, and biomarkers related to immunotherapy, such as PD-L1 (programmed death-ligand 1) and TMB (tumor mutational burden). In the meanwhile, we summarized and analyzed the progression-free survival (PFS) and the overall survival (OS) of patients with primary pulmonary nuclear protein of testis carcinoma treated with PD-1 (programmed cell death protein 1)/PD-L1 inhibitors and explored potential population that may benefit from immunotherapy. To the best of our knowledge, this is the first review on the exploration of the tumor microenvironment and immunotherapy effectiveness in primary pulmonary nuclear protein of testis carcinoma.

## 1 Introduction

The nuclear protein of testis (NUT) carcinoma is defined by the *NUT* (also known as *NUTM1*) gene rearrangement. The typical *BRD4-NUT* fusion gene is formed by the translocation rearrangement of the *NUT* gene on chromosome 15q and the *BRD4* gene on chromosome 19p (1), which accounts for at least 2/3 of NUT carcinoma (2). The *BRD3* gene on chromosome 9q (3)and the *NSD3* gene on chromosome 8p (4) are often fused with the *NUT* gene (5, 6). In recent years, with the rapid development of molecular technologies such as next-generation sequencing (NGS), rare fusion partners such as *ZNF532 *(7), *ZNF592* (8), *MXD4 *(9), *CIC *(10), *MGA *(11), *YAP1 *(12), *CHRM5 *(13)have been discovered one after another.

Since t (15; 19)(q15;p13) chromosome was first discovered in thymic carcinoma in 1991 (14), NUT carcinoma has always been regarded as a tumor inseparable from the midline structure, so it was called “NUT midline carcinoma”. However, as “NUT midline carcinoma” is successively discovered in structures or organs outside the midline, such as lungs (15), salivary glands (16, 17), kidneys (18, 19), adrenal glands (20) and soft tissues (18), the World Health Organization (WHO) has changed its name from “NUT midline carcinoma” to “NUT carcinoma” (21).

There is no gender difference in the prevalence of NUT carcinoma, which could be seen in any age group, but mainly children and young adults (5). The median age of NUT carcinoma patients is 23.6 years old (range=18days-80years) and is 30 years old (range=21years-68years) for primary pulmonary NUT carcinoma, respectively (6, 15).

NUT carcinoma is extremely aggressive, with rapid disease progression, poor treatment effect, high recurrence and mortality rate. The median OS (mOS) in NUT carcinoma is 6.5 months (6), while the mOS in primary pulmonary NUT carcinoma is only 2.2 months (15). Until the year of 2020, there were about 55 cases of primary pulmonary NUT carcinoma had been published in English (22). In order to have better treatment outcome and prognosis for patients with primary pulmonary NUT carcinoma, this article reviewed the latest diagnosis and treatment method, particularly the immunotherapy.

## 2 Etiology and Pathogenesis

### 2.1 Etiology

Due to the lack of cohort or large samples studies of primary pulmonary NUT carcinoma, the etiology remains unclear. Although some patients have a history of smoking, current evidences suggest that primary pulmonary NUT carcinoma occurs far more common among non-smokers (15). However, environmental factors and viral infections, such as Epstein-Barr virus, human papilloma virus, are not discovered to be related to primary pulmonary NUT carcinoma (13, 23, 24).

### 2.2 Pathogenesis

**Figure 1:**
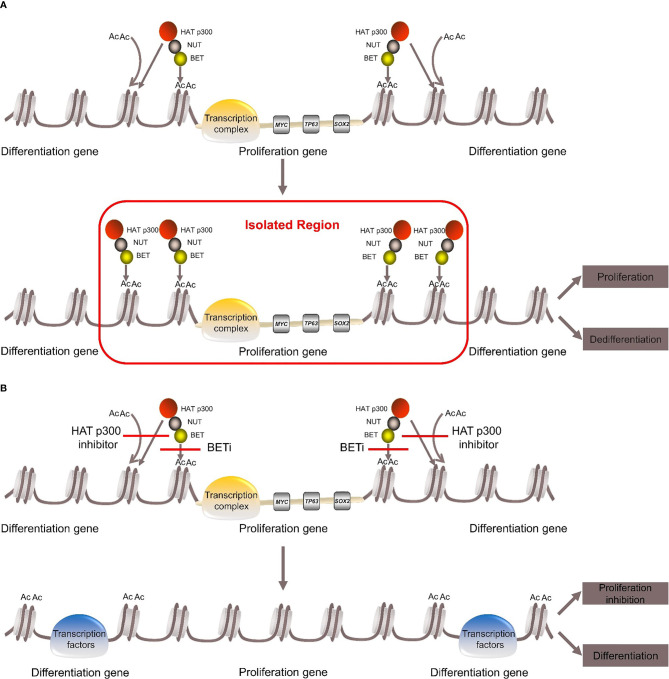
Pathogenesis of NUT carcinoma. **(A)** BRD4-NUT fusion oncoprotein binds to histone acetylated lysine residues in chromatin and recruits the histone acetyltransferase (HAT) p300. HAT p300 makes adjacent histones acetylated, which allows more BRD4-NUT fusion oncoproteins to bind to chromatin and recruit transcription factors to form transcription complex. The transcription complex is sequestered to regions of the chromatin that transcribe pro-proliferative and anti-differentiation genes, such as MYC, TP63, SOX. While it leads to the silencing of differentiation genes, thereby repressing differentiation and promoting proliferation. **(B)** With the use of BETi or HAT p300 inhibitor can induce differentiation and inhibit proliferation.

BRD4-NUT fusion oncoprotein (3, 5)contains parts of the BRD4 protein and the NUT protein. The BRD4 protein is a bromodomain and extraterminal domain (BET) protein. It binds BRD4-NUT fusion oncoprotein to histone acetylated lysine residues in chromatin *via* two bromodomains. The NUT protein can recruit the histone acetyltransferase (HAT) p300. It acetylates adjacent histones, which in turn allows more BRD4-NUT fusion oncoproteins to bind to chromatin and recruit transcription factors, such as positive transcription elongation factor b (P-TEFb) (25, 26). BRD4-NUT fusion oncoprotein sequesters histone acetyltransferases (HATs) and other transcriptional co-factors to the chromatin regions that transcribe pro-proliferative and anti-differentiation genes, such as *MYC*, *TP63*, *SOX2 (* 27–29). It also leads to the silencing of differentiation-promoting genes and hypoacetylation of the whole genome, thereby inhibiting differentiation and promoting proliferation (27, 30–34) (**Figure 1**).

The carcinogenic mechanism of BRD3-NUT fusion oncoprotein is similar to BRD4-NUT (35). The pathogenic mechanism of NSD3-NUT and ZNF532-NUT fusion oncoproteins was once thought to be similar to BRD4-NUT fusion oncoprotein (4, 7), but in recent years, it has been proposed that BRD4/BRD3, NSD3 and “Z4”, which consists of ZNF592, ZNF532, ZMYND8 and ZNF68, are combined into “BRD4-NUT complex”. Any component can directly bind to the NUT protein and recruit HAT p300. The complex then causes local chromatin lahyperacetylation, which promotes tumor growth and inhibition differentiation (8, 35).

## 3 Clinical Manifestation

The clinical manifestations of primary pulmonary NUT carcinoma are similar to those of lung cancer and are often closely related to tumor size, location, presence or absence of complications or metastases. Some patients are even asymptomatic, only founded in routine physical examination (36). The common symptoms included cough, chest pain, hemoptysis, wheezing, dyspnea, fever, etc., among which cough is the most common, and dyspnea caused by moderate to large pleural effusion is also frequently present (13, 15, 36).

## 4 Imaging Characteristics

**Figure 2:**
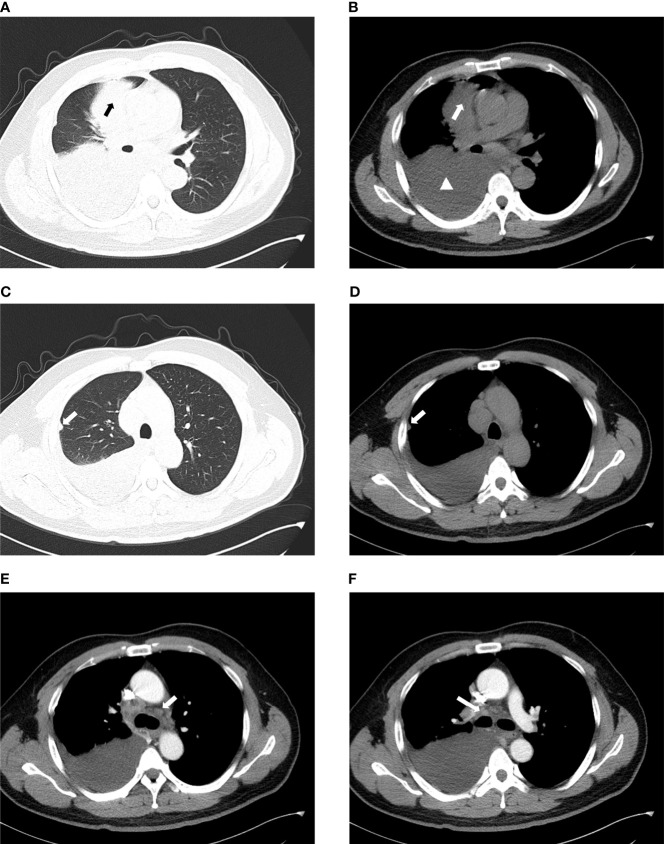
Imaging characteristics of chest CT. **(A, B)** The mass is located in the right upper lobe and is of central type with moderate to large pleural effusion on the right (arrow: mass, triangle: pleural effusion). **(C, D)** They show the ipsilateral pleural nodule but no involvement of the contralateral lung (arrow:pleural nodule). **(E)** Contralateral mediastinal lymphadenopathy. **(F)** Ipsilateral hilar lymphadenopathy.

The chest plain computed tomography (CT) scans showed irregular soft tissue density masses (**Figure 2**), mostly located in the lower lobe of the right lung (15, 37). Enhanced CT scans showed uneven enhancement of the masses. The lesions were large (maximum diameter of 12.7cm), mostly central and often fused with ipsilateral hilar and mediastinal lymphadenopathy (**Figure 2**). They often presented withobstructive atelectasis, ipsilateral pleural nodules and moderate or large pleural effusions (**Figure 2**). Supraclavicular, contralateral mediastinal, and subcarinal lymphadenopathy were also frequently present. Except for a small amount of pleural effusions on CT, there is no evidence of disease involvement in the contralateral lung (**Figure 2**). The extrathoracic metastatic sites were predominantly bones, with osteolytic changes on CT. Liver, adrenal gland, soft tissue involvement has also been reported.To our knowledge, case of brain metastasis in primary pulmonary NUT carcinoma has not been reported so far (13, 15, 38, 39).

PET/CT (positron emission tomography/CT) facilitated the staging and early detection of metastases. The 18F-flurodeoxyglucose (18F-FDG) of the pulmonary lesion was often highly concentrated (the standard uptake value (SUV) can be as high as 18.6). If the SUV in the center of the lesion decreased, it indicates necrosis of the lesion. In addition, PET/CT has good sensitivity for detecting bone metastases that cannot be detected by bone scintigraphy (15, 40).

Although the imaging findings of primary pulmonary NUT carcinoma show some characteristics, they are still not specific.

## 5 Diagnosis and Pathology

Since the etiology of primary pulmonary NUT carcinoma is still unclear, and the clinical manifestations, laboratory tests and imaging characteristics are non-specific, pathology is still the cornerstone of its diagnosis. Primary pulmonary NUT carcinoma was once considered as a subtype of lung squamous cell cancer (13, 41), but the WHO classified it as “other and unclassified carcinomas” in lung cancer in 2015 and 2021.

### 5.1 Gross Pathology

NUT carcinoma is extremely aggressive. Over 50% of patients have presented withorgans and/or lymph nodes metastases at the time of diagnosis (6, 41). Therefore, most patients cannot undergo surgery. In addition, the incidence of primary pulmonary NUT carcinoma is extremely low. Therefore, to date, there are few case reports on gross pathology. The gross examinations revealed an irregular solid tumor, which was not clearly demarcated from the surrounding tissues. Together with enlarged intralobar lymph nodes, they can cause airway compression and mucus obstruction. The cut surface of the tumor was brown and white or white and may be accompanied by hemorrhage and necrosis (22, 37, 42–44).

### 5.2 Cytology

#### 5.2.1 Pulmonary Mass and Lymph Node Needle Aspiration Cytology

**Figure 3:**
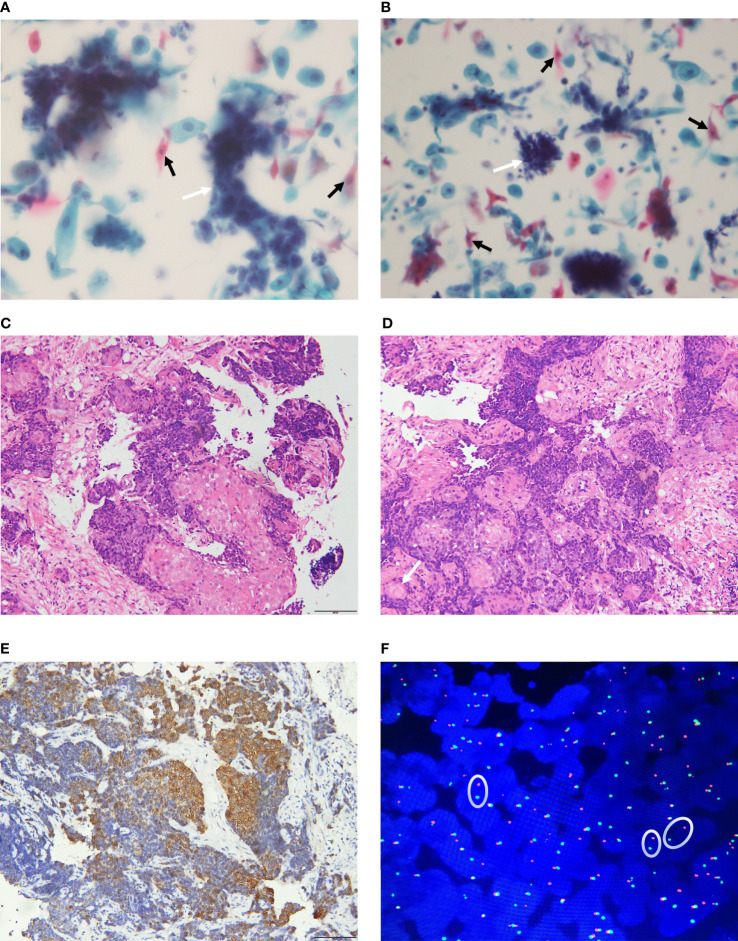
Pathological features of NUT carcinoma. **(A, B)**: The tumor cells are nested or sheet-like arrangement (white arrow). The nuclei are large and hyperchromatic. Tumor cells of squamous epithelial differentiation can be seen (black arrow). **(C, D)** (H&E staining): The tumor is poorly differentiated and shows infiltrative growth. The tumor cells are irregular nest-like, polygonal, large nucleus and heteromorphic. There are abrupt squamous epithelial differentiation and keratinized bead (arrow). **(E)** (IHC): CK5/6 (+) **(F)** (FISH): Dual-color split probes targeting both sides of the NUT gene breakpoint are seperated (oval).

The tumor cells were medium in size and relatively monotonous,with sheet-like arrangement. The nuclei were large and hyperchromatic, which could be round, ovoid or irregular (45–47) (**Figures 3A, B**). Scattered bare nuclei were also observed (46, 47). The nucleoli was prominent and nuclear chromatin was fine to granular (45, 47, 48). Mitoses and apoptotic nuclei were frequent (46, 47).

#### 5.2.2 Pleural Effusion Cytology

Monomorphic tumor cells were arranged in isolation or clusters, with irregular nuclear contours, prominent nucleoli and coarse chromatin granules (49).

### 5.3 Histology

Most primary pulmonary NUT carcinoma were poorly differentiated or undifferentiated neoplasm (50–52). The tumors had invasive growth and could invade the bronchial walls or blood vessels (53, 54). The tumour cells were medium in size, relatively monotonous and could be round, epithelioid or polygonal. They were arranged in nested or sheeted pattern (39, 49) and could be accompanied by obvious proliferation of interstitial connective tissues (42, 44, 53, 54). Neutrophil infiltration and necrosis may occur in the background (44, 47–51, 53). The nuclei may be round, oval or irregular, with prominent nucleoli, open chromatin, which were granular to coarse. The cytoplasm was scarce and the nucleocytoplasmic ratio was high (15, 22, 44, 48, 50, 52, 55). Mitotic figures were evident (22, 50, 51, 53, 54). The representative pathological changes of primary pulmonary NUT carcinoma are abrupt differentiation of squamous epithelial and keratinized beads (**Figures 3C, D**) (51, 54).

### 5.4 Immunohistochemistry

Most primary pulmonary NUT carcinoma cases were positive for cytokeratins (AE1/AE3, CAM5.2, CK5/6, pan-cytokeratin were more common) (**Figure 3E**), P63, P40, and NUT (37, 56, 57). TTF-1 and a variety of neuroendocrine markers were mostly negative (13, 39, 55, 58). Ki-67 (13, 37, 45, 48), EMA (22, 47, 54), and C-MYC (58) were positive in some cases. Clinicians and pathologists lack relevant knowledge of primary pulmonary NUT carcinoma. Relatively speaking, there is no consistent selection criteria for immunohistochemical staining markers for primary pulmonary NUT carcinoma. Therefore, the results of immunohistochemical staining varied widely among the literatures. Using highly specific anti-NUT monoclonal antibody C52 for Immunohistochemical staining, the sensitivity of the diagnosis of NUT carcinoma is 87% and the specificity is 100% (59), but the fusion partner of the NUT gene cannot be identified.

### 5.5 Molecular Pathology

In addition to classic cytogenetic karyotype analysis (14, 60), fluorescence *in situ* hybridization (FISH) (22, 50, 61), reverse transcription polymerase chain reaction (RT-PCR) (52, 55, 57) and NGS, including RNA sequencing (4, 7), Archer FusionPlex (8, 18) as well as whole-genome sequencing (WGS) (62) are effective diagnostic methods (**Table 1**). However, various diagnostic methods have certain limitations (23): (1) Cytogenetic was the traditional method for discovering classic t (15;19)(q14;p13.1) fusion. It was undoubtedly the “gold standard”, but its high cost and the need for fresh live tumor greatly limited its application. (2) RT-PCR: Due to the need to use known specific primers, it is impossible to identify rare, unknown, and non-classical fusion genes, which can easily lead to missed diagnosis or misdiagnosis. (3) Archer FusionPlex and whole-genome sequencing: Although it can identify any fusion partner of NUTM1, detect gene mutations and TMB, it is costly and requires a large amount of tumor tissues. (4)FISH: The NUTM1 dual-color translocation rearrangement FISH probes targeting the NUT breakpoint and the fusion-partner breakpoint can detect the fusion of corresponding genes(two separate probes are close to each other). They can confirm the diagnosis of NUT carcinoma and the type of known fusion partners (30, 50). Moreover, the NUTM1 dual-color split probes were also used for diagnosis (**Figure 3F**) (13, 58, 61, 63), but the fusion partners could not be identified. False negative FISH results have been reported (59, 64), so it is recommended to use a probe spanning NUTM1 (59) or a combination of conventional IHC and C52 antibody for diagnosis. The sensitivity of FISH combined with C52 IHC in diagnosing NUT carcinoma can reach 100% (59, 65).

**Table 1:** Various diagnostic methods of molecular pathology.

Diagnostic Methods	Advantages	Limitations
Cytogenetic	Gold standard	High cost The need for fresh live tumor
RT-PCR	Fast	The need for using known specific primers Impossible to identify rare, unknown, and non-classical fusion genes
NGS	Identify any fusion partner Detect gene mutations and TMB	High cost The need for a large amount of tumor tissues
FISH	Can be used on multiple sample types (frozen tumor, air dried, or FFPE)	Fusion partners cannot be identified using the NUTM1 dual-color split probes False negative*

*The sensitivity of FISH combined with C52 IHC in diagnosing NUT carcinoma can reach 100%.

## 6 Differential Diagnosis

Primary pulmonary NUT carcinoma is very aggressive and is undifferentiated or poorly differentiated. Early diagnosis is of great significance for prognostic judgment. Histology shows squamous differentiation and formation of keratinized beads. Immunohistochemical staining is mostly positive for cytokeratins, p63 and P40. So it is easily misdiagnosed as poorly differentiated squamous cell carcinoma. However, primary pulmonary NUT carcinoma shows abrupt squamous differentiation and immunohistochemical staining with anti-NUT monoclonal antibody is positive. Both primary pulmonary NUT carcinoma and small cell lung cancer (SCLC) show scarce cytoplasm and high Ki-67 proliferation index, but the latter has no obvious nucleoli and no focal squamous cell differentiation. Immunohistochemical staining is positive for chromogranin and synaptophysin, negative for NUT (46, 63). Differentiation of poorly differentiated lung adenocarcinoma and primary pulmonary NUT carcinoma mainly relies on immunohistochemistry. Immunohistochemical staining of adenocarcinomais positive for TTF-1 and Napsin A, negative for p40, p63 and NUT. Patients with poorly differentiated or undifferentiated lung tumor, especially those who are young, nonsmokers or lack of other high-risk factors, should be alert to primary pulmonary NUT carcinoma. In particular, patients with rapid disease progression, extensive invasions and poor response to initial treatment, anti-NUT monoclonal antibody immunohistochemical staining should be performed as soon as possible, combined with FISH if necessary.

## 7 Therapy Strategies

The treatment of primary pulmonary NUT carcinoma mainly included surgery, chemotherapy and radiotherapy. In recent years, targeted therapy, antiangiogenic therapy and immunotherapy have also been reported (13, 57, 66). In addition, novel targeted drugs such as BET inhibitor (BETi), p300/CBP HAT inhibitor, histone deacetylase inhibitor (HDACi) and dual HDAC/PI3K inhibitor are also considered as potential treatments (32, 58, 67–72).

### 7.1 Surgery

Surgery is the first choice for almost all solid malignant tumors. However, due to the aggressive nature of NUT carcinoma, most patients have missed the best timing when they were diagnosed and even lost the opportunity for surgical treatment, which greatly reduces the rate of radical operation. However, early radical operation can still significantly improve the PFS and OS of NUT carcinoma (2, 6, 41). It has been reported (66, 73) that the disease free survival (DFS) of a patient staged as T1bN0M0 with primary pulmonary NUT carcinoma and treated with first-line radical surgery and adjuvant chemotherapy is up to 30 months, far exceeding 2.2 months of mOS, as well as long-term survival. One patient at T3N1M0 stage underwent radical lobectomy and regional lymphadenectomy, with adjuvant etoposide and platinum combined with bevacizumab. As of the publication of the literature, DFS has reached 10 months (66). It is suggested that radical surgery and adjuvant chemotherapy (combined with antiangiogenic therapy in locally advanced-stage patients) in early-stage patients can significantly improve the prognosis.

### 7.2 Chemoradiotherapy and Chemotherapy

Up to now, most of the cases who had lost the opportunity for surgery have received chemoradiotherapy or chemotherapy. In patients receiving chemoradiotherapy, the shorter OS was 2 months to 4 months (39, 55), and the longer OS reached 148weeks (47, 54, 57, 61). The OS was significantly higher than the mOS of primary pulmonary NUT carcinoma. It is consistent with the conclusion that radiotherapy can improve the prognosis of NUT carcinoma (2, 6, 41). For patients receiving chemotherapy, after a short-term response or stable disease, they often progressed rapidly (13, 57). According to the literatures, the longest PFS was 5 months (74) and the longest OS was 13 months (75). However, until now, no chemotherapy regimen with definite therapeutic effect has been recognized.

Therefore, for patients who have lost the opportunity for surgery, chemoradiotherapy may significantly benefit patients, but the exact effective regimens still need to be further explored.

### 7.3 Targeted Therapy

Because primary pulmonary NUT carcinoma was often misdiagnosed as undifferentiated or squamous cell lung cancer, driver genes were rarely detected. As far as we know, there are few reports of EGFR mutation cases (13, 63) and none of ALK and ROS1 rearrangements cases have been reported. Xiaohong Xie et al. reported a patient with EGFR exon 19 deletion, who received gefitinib in the second-line (the PFS was 2 weeks) apatinib in the third-line (the PFS was 1 month). The OS was 4.1 months (13). Although the results were not satisfactory, it provided a possible idea for the treatment of primary pulmonary NUT carcinoma.

Clinical trials of BET inhibitors have been carried out in recent years. Although several BETi have showed certain anti-NUT carcinoma activity, the efficacy of single-agent was limited (76, 77). The response rate in NUT carcinoma was only 20-30% (67, 68). In preclinical trials, p300/CBP HAT inhibitor has been shown to have inhibitory effects in NUT carcinoma. The combination of p300/CBP HAT inhibitor and BETi even has synergistic effects (32, 67, 68). The anti-NUT carcinoma activity of HDACi was also confirmed in animal models and two cases of NUT carcinoma in children (69, 70). Moreover, the anti-NUT carcinoma activity of CUDC-907 (dual HDAC/PI3K inhibitor) has been demonstrated *in vitro* and animal models, even better than HDACi (58, 71, 72).

In general, targeted therapy significantly improves the prognosis of lung cancer with targetable driver oncogenes. If patients with primary pulmonary NUT carcinoma have targetable driver oncogenes, targeted therapy may be a potential treatment option. In addition, novel targeted drugs have appeared in the treatment of NUT carcinoma and are worth looking forward to in the future.

### 7.4 Immunotherapy

Since the Food and Drug Administration (FDA) first approved Nivolumab for the treatment of advanced lung cancer in 2015, immunotherapy has developed so rapidly to significantly improved the prognosis.

#### 7.4.1 The Main Mechanism of PD-1 or PD-L1 Inhibitors

**Figure 4:**
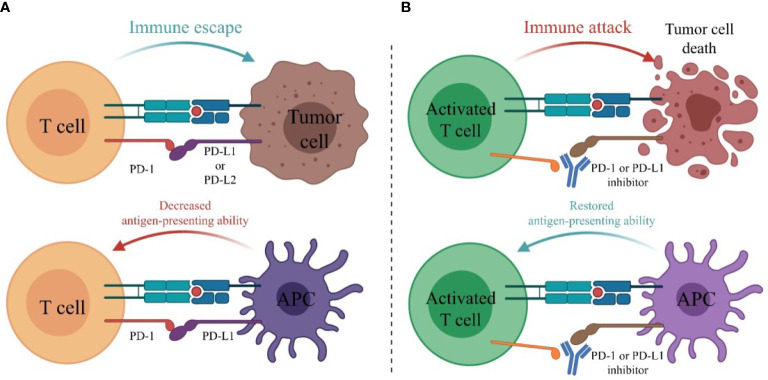
The main mechanism of PD-1 or PD-L1 inhibitors. **(A)** PD-1 is expressed on the surface of T cells. Its ligands are PD-L1 or PD-L2 on the surface of tumor cells and PD-L1 on the surface of antigen-presenting cells (APCs). The combination of PD-1 and its ligand inhibits the activation of T cells, leading to immune escape of tumor cells. **(B)** PD-1 or PD-L1 inhibitors block the binding of PD-1 to its ligands and restore the immune killing of tumor cells.

**Figure 5:**
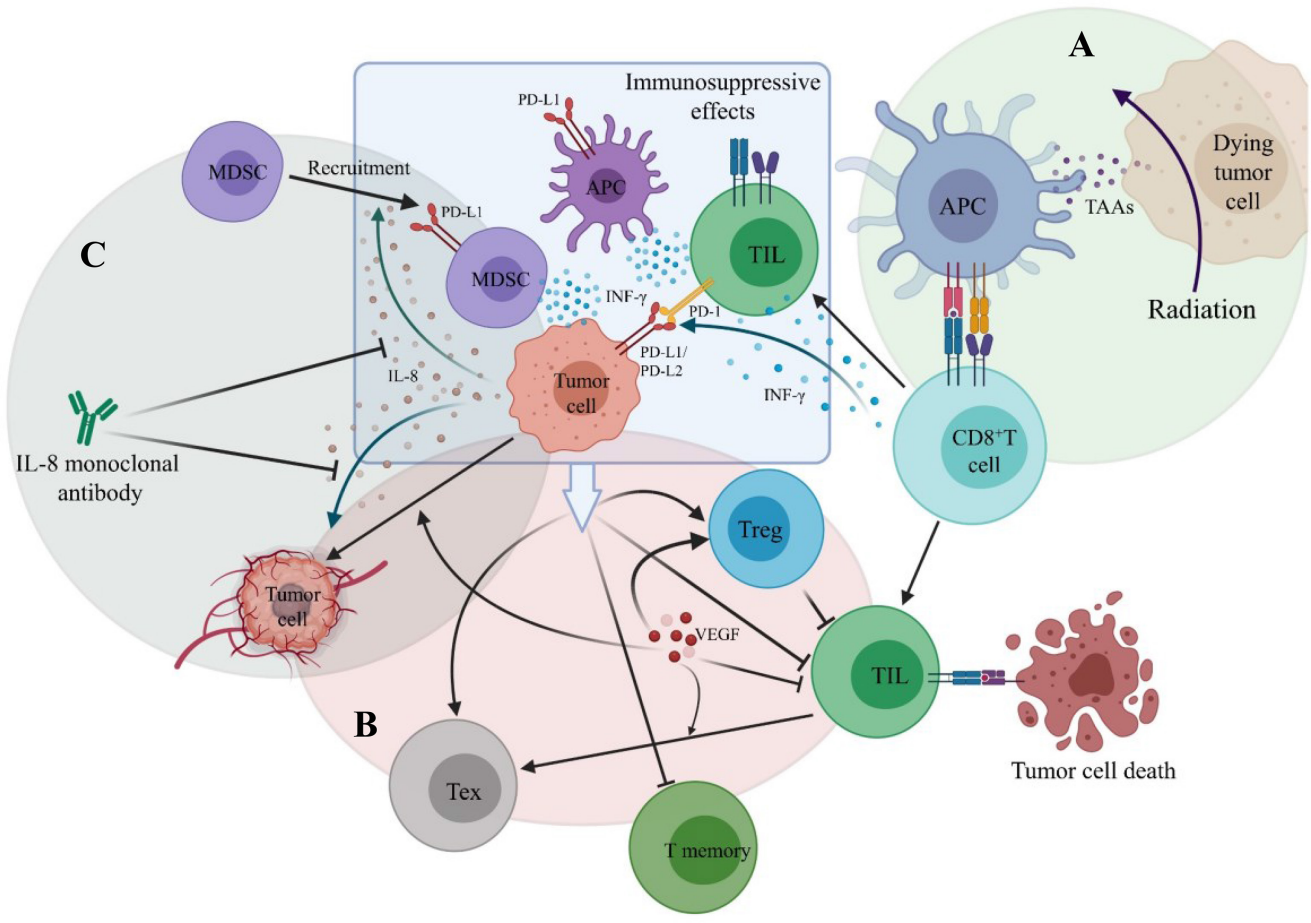
The main mechanisms of combination therapy with PD-1/PD-L1 inhibitors. **(A)** Radition results in tumor cell death. Tumor-associated antigens (TAAs) are released and antigen-presenting cell is activated. CD8+ T cell is then primed by binding to APC. **(B)** VEGF stimulates angiogenesis, promotes the infiltration of T regulatory cell (Treg), decreases TIL infiltration and promotes the formation of T exhausted cell (Tex). **(C)** Tumor-derived IL-8 can promote tumor angiogenesis and recruit MDSC to suppress anti-tumor immune responses.

PD-1 is expressed on the surface of T cells. Its ligands are PD-L1 or PD-L2 on the surface of tumor cells and PD-L1 on the surface of antigen-presenting cells (APCs), mainly including dendritic cells (DCs) and macrophages. The combination of PD-1 and its ligand can activate the PI3K-Akt-mTOR pathway in tumor cells, resulting in a decrease in T effector cells and T memory cells with immunostimulatory effects and an increase in T regulatory cells (Treg) and T exhausted cells (Tex) with immunosuppressive effects, leading to immune escape of tumor cells (78). PD-1 inhibitors or PD-L1 inhibitors can block the binding of PD-1 to its ligand and restore the immune killing of tumor cells (**Figures 4**, **5**).

#### 7.4.2 The Present Condition of Immunotherapy in Primary Pulmonary NUT Carcinoma

In recent years, cases of primary pulmonary NUT carcinoma receiving immunotherapy have also been reported (see **Table 2** for clinicopathological features, treatments methods and survival results). The immunotherapy drugs mentioned in the reported literature for patients with primary pulmonary NUT carcinoma included PD-1 inhibitors (Nivolumab, Pembrolizumab) or PD-L1 inhibitor (Atezolizumab). The vast majority of patients received immunotherapy as second-line or beyond (subsequent) treatment.

**Table 2:** Clinicopathological features, immunotherapy and outcomes.

Patients	Age(years)/Gender	Initial diagnosis	TNM staging⁑	PD-L1	TMB (muts/Mb)	Treatment1	PFS1	Treatment2	PFS2	Treatment3	PFS3	Treatment4	PFS4	Treatment5	PFS5	OS	NUTM1-fusion	Reference
1	23/M	Mucinous epithelial carcinoma	IVA (T3N2M1b)	ND	11.55	Operation	1 mo	Atezo	2w	–	–	–	–	–	–	1.5 mo	ND	(13)
2	30/F	SCC	IVA (T4N3M1b)	ND	ND	TP*	6w	T	3w	T + Nivo	3w	–	–	–	–	3 mo	ND	(13)
3	74/M	NC	IVA (T3N3M1a)	ND	High	RT	1 mo	Pembro	21w	Support care	13 mo	–	–	–	–	19.5mo	ND	(13)
4	58/F	NC	IIIC (T4N3M0)	ND	73.81	Cet+RT+DP	18w	Support care	18 mo	Pembro+Cet	3mo	Pembro+Oxaliplatin	3w	Support care	–	26.7mo	ND	(13)
5	31/F	NC	IIIA (T4N1M0)	ND	1.75	DP+ Cet	6w	T+Gemcitabine+Nivo	2w	Nivo+Iri+Platinum	3w	Pembro+ Carboplatin+ RT	3mo	Support care	–	12mo+	CHRM5	(13)
								Pembro+ T+Gemcitabine+Nivo	4w									
6	45/M	NC	IIIA	70%	ND	AC†+Pembro	NA	NA	NA	NA	NA	NA	NA	NA	NA	12mo+	ND	(36)
7	48/F	SCC	IIIA	0%	ND	Genexol+ Carboplatin + Pembro, Lobectomy+Pembro	NA	NA	NA	NA	NA	NA	NA	NA	NA	12mo+	ND	(36)
8	31/M	NA	IV	ND	ND	CRT,Atezo**	NA	NA	NA	NA	NA	NA	NA	NA	NA	2.2mo	NSD3	(39)
9	53/M	NA	III	ND	ND	CRT,Nivo,HDAC inhibitor**	NA	NA	NA	NA	NA	NA	NA	NA	NA	11.6mo	BRD3	(39)
10	57/M	PD SCC	IVA (T4N3M1a)	ND	ND	DP	6w	Nivo	6w	–	–	–	–	–	–	4mo	ND	(49)
11	31/F	SCC	IA (T1bN0M0)	10%(SP263)	ND	Lobectomy adjuvant TP§	30mo (DFS)	Observed off-treatment	7mo	Nivo	29 mo	Nivo	13mo	NA	NA	79mo+	ND	(73)
12	34/F	NC	IVB	Negative	ND	TP*	5mo	Pembro	Lost	–	–	–	–	–	–	Lost	ND	(74)

CD34 expression and MSI status were not detected in all patients.

^⁑^Using IASLC Eighth Edition of the TNM Classification for Lung Cancer to re-staging the cases with detailed data, while directly quoting the staging in the original literatures for the cases with insufficient data.

*nab-Paclitaxel(T)+Carboplatin, §Paclitaxel+Carboplatin, †AC: Pemetrexed+Carboplatin, **The treatment protocol was not described in detail.

Atezo, Atezolizumab; Cet, cetuximab; CRT, chemotherapy+radiotherapy; F, female; Iri, irinotecan; Lost, lost follow-up; mo, months; M, male; NA, data not available; NC, NUT carcinoma; ND, not done; Nivo, Nivotuzumab; PD, poorly differentiated; Pembro, Pembrolizumab; RT, radiotherapy; SCC, squamous cell carcinoma; w, weeks.

The best OS of patients stage IVA who underwent surgery or chemoradiotherapy in the first-line and Atezolizumab in the second-line was only 2.2 months (13, 39), which is similar to the mOS of primary pulmonary NUT carcinoma. Atezolizumab does not seem to improve the prognosis of advanced patients.

One patient of stage IA (Patient 11 in **Table 2**) was reported to receive radical surgery and adjuvant chemotherapy in the first-line and Nivolumab monotherapy in the third-line. The PFS reached 29 months and the OS was at least 79 months (73). So far, this patient had the longest survival time among the primary pulmonary NUT carcinoma patients retrieved in the literatures, who had received immunotherapy. Two patients received the combination treatment including Pembrolizumab as the first-line treatment, the OS had exceeded 12 months (36), suggesting that first-line use of Pembrolizumab may improve the prognosis. The OS of two non-surgical patients staging III who had previously received chemoradiotherapy and then received Nivolumab or Pembrolizumab was significantly prolonged [11.6 months (39), 26.7 months (13) respectively]. The OS of one non-surgical patient staging IVA who received chemotherapy in the first-line and Nivolumab in the second-line (The PFS was 6 weeks) was 4 months (49), and that of one non-surgical patient staging IVA who received radiotherapy in the first-line and Pembrolizumab in the second-line (The PFS was 21 weeks) was 19.5 months (13). Therefore, it seems that patients, whose tumor are unresectable or not fit for surgery, are likely to benefit more from Pembrolizumab than Nivolumab, especially those who ever received chemoradiotherapy or radiotherapy. This seems to be consistent with the view that radiotherapy combined with immunotherapy can achieve a benefit in overall survival in lung cancer (79, 80). The probable mechanisms (**Figure 5**) are that after receiving radiotherapy, dead tumor cells release tumor-associated antigens (TAAs) and inflammatory cytokines. Dendritic cells recognize them and are activated, promoting antigen presentation to cells of the immune system, and CD8^+^ T cells are then primed and recruited to the tumor site, thereby killing tumor cells (81, 82). In the meanwhile, radiotherapy can upregulate the expression of PD-L1 on tumor cells *via* IFN-γ released by CD8^+^ T cells and the PD-1 levels on CD8^+^ tumor infiltrating lymphocytes (TILs) (78, 82), which enhance the effect of PD-1 inhibitors. Although the optimal PFS of PD-1 inhibitors in non-surgical patients was only 21 weeks, which was similar to that of chemotherapy. However, compared with chemotherapy, the OS of non-surgical patients was significantly prolonged.

#### 7.4.3 The Recommendations for the Application of Immunotherapy in Primary Pulmonary NUT Carcinoma

(1) Atezolizumab does not seem to improve the prognosis of advanced patients. (2) For early-stage patients, PD-1 inhibitors used in the second-line, after radical surgery and adjuvant chemotherapy in the first-line, could distinctly prolong the PFS and the OS. (3) For advanced-stage patients or whose tumor is surgically unresectable, if they ever received chemoradiotherapy or radiotherapy, PD-1 inhibitors could significantly prolong the OS, and Pembrolizumab may be better than Nivolumab. (4) Combination therapy with Pembrolizumab or Pembrolizumab monotherapy in first-line may improve the OS.

#### 7.4.4 PD-1 Inhibitor in Combination With BETi

In mouse models and a wide variety of human tumor cell lines, BETi inhibited constitutive and IFN-γ induced PD-L1 expression on tumor cells and tumor-associated dendritic cells and macrophages, which correlated with an increase in the activity of TILs (83, 84). Moreover, the combination of PD-1 inhibitor and BETi caused synergistic effects in mice (83, 84).

Therefore, patients with primary pulmonary NUT carcinoma may benefit from the combination treatment of PD-1 inhibitor with BETi. However, this inference needs more basic researches and clinical trial results to testify.

## 8 Targeting the Tumor Microenvironment

### 8.1 Tumor Angiogenesis and VEGF (Vascular Endothelial Growth Factor)

Tumor angiogenesis is closely related to the growth and metastasis of cancer. In addition to stimulating endothelial cell growth and angiogenesis, VEGF can also decrease TILs infiltration, promote the infiltration of Tregs (**Figure 5**), and increase the expression of inhibitory receptors contributing to CD8^+^ TILs exhaustion (85, 86). As a antigen of vascular endothelial cell, after immunohistochemical staining, CD34 can be used as a marker of angiogenesis to count microvessel density (MVD) and the degree of neoangiogenesis (87, 88). High expression of CD34 or high MVD is closely correlated to the tumor progression and poor prognosis (88–90). Moreover, it has been indicated that the inhibition of CD34 expression may repress neoangiogenesis, tumor growth and invasion (91).

### 8.2 The Relationship Between the Prognosis and CD34 Expression in Primary Pulmonary NUT Carcinoma

Cases of primary pulmonary NUT carcinoma involving CD34 expression have also been successively reported in recent years (Clinicopathological features, treatments, and survival outcomes are shown in **Table 3**). The best OS for non-surgical patients of stage IV with primary pulmonary NUT carcinoma, with CD34-positive staining, was at least 100 weeks (61, 92). The worst OS in stage IIIC non-surgical patients with CD34-negative staining was 2 months (93), while the best OS in stage IV non-surgical patients with CD34-negative staining was 148 weeks (52, 61). Therefore, the published literatures have not found significant correlation between CD34 expression and the prognosis of patients with primary pulmonary NUT carcinoma, which needs more patients data for further evaluation.

**Table 3:** Clinical features, immunohistochemistry CD34 staining, treatments and outcomes.

Patients	Age(years)/Gender	Initial diagnosis	TNM staging⁑	CD34	PD-L1	TMB (muts/Mb)	MSI	Treatment1	PFS1	Treatment2	PFS2	OS	NUTM1-fusion	Reference
1	36/M	Nonseminomatous Primary mediastinal germ cell tumor	T3N3Mx	Negative	ND	ND	ND	I/E/Cisplatin	2cylces	RT,BETi	2cylces	2mo	BRD4	(30)
2	34/M	Spindle cell neoplasm	IV	Positive	80%	ND	ND	CRT(Genexol/Carboplatin),Pembro	Lost	–	–	Lost	ND	(36)
3	48/M	NC	T4N2M0 (IIIB)	Negative	ND	ND	ND	Radical operation	2mo	Palliative RT (right scapula)	NA	6mo	ND	(43)
4	66/M	NC	T4NxM1c	Negative	ND	ND	ND	Palliative care	NA	NA	NA	1mo	ND	(54)
5	14/M	Unspecified sarcoma or undifferentiated arcinoma	T4NxMx	Negative	ND	ND	ND	IRS III regimen 36(VCR/E/CTX/Cisplatin)	NA	I/Carboplatin/E, Lobectomy+ I/Carboplatin/E	NA	12mo	BRD4	(55)
6	7/F	Undifferentiated SCC	IV	Negative	ND	ND	ND	CRT (TP)	NA	NA	NA	4mo	BRD4	(55)
7	16/F	PD carcinoma	IV	Positive	ND	ND	ND	CRT	NA	NA	NA	100w+	ND	(61)
8	16/M	SCC	IVB	Negative	ND	ND	ND	CRT	NA	NA	NA	148w	BRD4	(61)
9	26/M	NA	T4NxMx	Negative	ND	ND	ND	E	NA	NA	NA	5mo	ND	(63)
10	69/M	NA	T2NxMx	Negative	ND	ND	ND	E	NA	NA	NA	6mo	ND	(63)
11	69/M	Neuroendocrine carcinoma with adenocarcinoma	T3N1M0 (IIIA)	ND	Negative (22C3)	1.6	Stable	Radical operation adjuvant EP(1^th^-3^th^ cycle)/EC(4^th^cycle) and Avastin	10mo+(DFS)	–	–	Not reached	ND	(66)
12	17/M	NC	IVB	Positive	ND	ND	ND	SSG IX	NA	NA	NA	5mo	ND	(92)
														
13	23/F	Non-Hodgkin’s lymphoma	T4NxMx	Negative	ND	ND	ND	DP,Vorinostat‡	NA	NA	NA	2mo	BRD4	(93)

^⁑^Using IASLC Eighth Edition of the TNM Classification for Lung Cancer to re-staging the cases with detailed data, while directly quoting the staging in the original literatures for the cases with insufficient data.

‡Vorinostat, an HDAC inhibitor; CRT, chemotherapy+radiotherapy; CTX, Cyclophosphamide; E, Etoposide; F, female; I, Ifosfamide; Lost, lost follow-up; mo, months; M, male; NA, data not available; NC, NUT carcinoma; ND, not done; PD, poorly differentiated; Pembro, Pembrolizumab; RT, radiotherapy; SCC, squamous cell carcinoma; VCR, Vincristine; w, weeks.

### 8.3 The Rationale for Anti-VEGF Therapy and the Present Condition of Anti-VEGF Therapy

By inhibiting VEGF-mediated suppression of dendritic cells maturation, Bevacizumab can trigger and activate T-cell response (94). In addition, Bevacizumab downregulates PD-1 expression on CD8^+^ TILs and results in an increased infiltration of T cells into the tumor by normalising the tumor vasculature (95–99). In the meanwhile, Bevacizumab reprogrammes the tumor microenvironment by inhibiting the activity of myeloid derived suppressor cells (MDSCs) and Treg cells (98, 100–102). One patient with primary pulmonary NUT carcinoma (patient 11 in **Table 3**, TNM staging:T3N1M0-IIIA) received radical lobectomy, regional lymphadenectomy and adjuvant EP (etoposide and platinum) combined with Bevacizumab (Avastin) for 4 cycles. The DFS had reached at least 10 months by the time of literature publication (66). Regrettably, the expression of CD34 was not tested in the patient. In addition, two patients with orbital NUT carcinoma, who had undergone operation, received anlotinib in third-line. The OS was beyond 15 months and 8 months, respectively (103, 104).

According to IMpower150 research, compared with Bevacizumab combined with paclitaxel plus carboplatin, Atezolizumab plus Bevacizumab combined with paclitaxel plus carboplatin significantly improved the PFS and OS in lung cancer, regardless of PD-L1 expression. Therefore, we consider that anti-VEGF therapy combined with PD-1/PD-L1 inhibitor plus chemotherapy may be beneficial to patients with primary pulmonary NUT carcinoma.

In summary, anti-VEGF therapy may be a potential treatment for primary pulmonary NUT carcinoma, especially in postoperative adjuvant treatment and combination application with PD-1/PD-L1 inhibitor (particularly Pembrolizumab) plus chemotherapy.

### 8.4 Immunosuppressive Effects of Interleukin-8 (IL-8)

As the correlation between IL-8 and tumor is being investigated in full swing, the impacts of tumor-derived IL-8 on the tumor microenvironment have been clearer (105, 106). It has been confirmed that tumor-derived IL-8 can promote tumor angiogenesis, recruit MDSCs to suppress anti-tumor immune responses (**Figure 5**) and maintain the epithelial mesenchymal transition phenotype of tumor cells, thereby participating in the proliferation and metastasis of tumor cells (107–110).

### 8.5 IL-8 and Prognosis on Immunotherapy

Low baseline serum IL-8 (sIL-8) level and early decline of sIL-8 are in correlation with the benefit from immune checkpoint inhibitors as well as better cancer prognosis. The mOS of patients undergoing Nivolumab with low (<23 pg ml^−1^) baseline sIL-8 levels was as 2 to 3 times as high (≥23 pg ml^−1^) baseline sIL-8 levels. The decline of sIL-8 levels, 2-4 weeks after starting treatment, was significantly correlation with response to PD-1 inhibitor. The mOS of patients receiving PD-1 inhibitor with sIL-8 decrease over baseline was not reached, while that of those with sIL-8 increase over baseline was 8 months. Besides, the decline of sIL-8 can help judge immunotherapy pseudoprogression when imaging evaluation is progressive disease. In addition, levels of sIL-8 are not correlated with the PD-1 and PD-L1 expression (111–113).

Therefore, based on sIL-8 levels, we will consider whether to choose immune checkpoint inhibitor for treatment and estimate the prognosis in primary pulmonary NUT carcinoma.

### 8.6 IL-8 Monoclonal Antibody

It has been demonstrated that IL-8 monoclonal antibody can suppress tumor angiogenesis (**Figure 5**), significantly reduce tumor size *in vitro* and animal models (114, 115), and decrease recruitment of MDSCs to the tumor (116). Even more exciting is that a Phase I trial of patients with metastatic or unresectable solid tumors showed that sIL-8 significantly decreased on the third day of IL-8 monoclonal antibody monotherapy. Among 73% of patients, their disease was stable, with the median treatment duration of 24 weeks (117). For patients with higher sIL-8 level in primary pulmonary NUT carcinoma, it is good news. The combination of IL-8 monoclonal antibody with immune checkpoint inhibitor or anti-VEGF therapy, may be new directions for the treatment in the future. This is consistent with the results of previous laboratory studies (116, 118). We look forward to further clinical trials results of IL-8 monoclonal antibody in the treatment of tumor.

### 8.7 Neutrophil/Lymphocyte Ratio (NLR) and Immunotherapy

In multiple studies of patients receiving PD-1/PD-L1 inhibitors, it was found that lower baseline NLR in the blood was associated with better response, PFS and OS. The cutoff value of NLR was 5 in most of studies. In addition, the decline of NLR during treatment often indicated that immunotherapy was effective (119–122).

Therefore, baseline NLR levels and dynamic NLR changes may also be biomarkers for determining whether patients with primary pulmonary NUT carcinoma can benefit from immunotherapy.

## 9 Prognosis

One study (6) that included 124 patients of NUT carcinoma found that the mOS of nonthoracic primary NUT carcinoma was significantly better than that of thoracic primary NUT carcinomas, and patients with BRD4-NUT fusion had worse mOS than those with BRD3-NUT or NSD3-NUT fusion. It was consistent with the conclusions of previous reports (41, 61). The mOS of NUT carcinoma is 6.7 months (41), whereas the mOS of primary pulmonary NUT carcinoma is only 2.2 months (15).

It is well known that PD-L1 expression level has a certain correlation with the benefit from immunotherapy. Few cases on PD-L1 expression level of primary pulmonary NUT carcinoma have been reported, and PD-L1 TPS (Tumor Proportion Score) varied from 0-80% (36, 73). However, due to the uneven quality of cases data, it is impossible to precisely evaluate the relationship between PD-L1 expression level and the benefit from immunotherapy or prognosis in primary pulmonary NUT carcinoma. In addition, TMB, microsatellite instability (MSI) and DNA mismatch repair (MMR) do not seem to be associated with the prognosis (13, 66, 123).

## 10 Conclusion

Although primary pulmonary NUT carcinoma is rare, it is recognized gradually in recent years with the application and development of immunohistochemistry and molecular pathology. However, there is still a lack of general understanding and clear awareness in clinical practice. Patients with poorly differentiated or undifferentiated lung tumors who are youthful, nonsmokers, and lack of other high-risk factors for lung cancer, particularly those with sudden squamous epithelial differentiation with or without keratosis beads formation, should be highly alert to primary pulmonary NUT carcinoma. In addition, for patients with central lung mass, moderate to large pleural effusion ipsilaterally, extensive infiltrating lesions, rapid disease progression and poor response to initial therapy, anti-NUT monoclonal antibody immunohistochemical staining should be performed as soon as possible, if necessary, combined with FISH (using the NUTM1 dual-color translocation rearrangement probes and the sensitivity of combining FISH with C52 IHC for diagnosing NUT carcinoma can reach 100%), NGS or whole genome sequencing.

Many attempts have been made in the treatment of primary pulmonary NUT carcinoma in recent years, but a standard and consistently effective treatment has not yet been established.

For early-stage or locally advanced patients, radical surgery and adjuvant chemotherapy (for locally advanced-stage patients, combined with anti-VEGF therapy, in the meanwhile) could distinctly prolong the PFS and the survival time. The OS can be further extended using PD-1 inhibitor as the second-line treatment.

Patients who have lost the opportunity for surgery can significantly benefit from chemoradiotherapy. For advanced-stage or patients with tumors unresectable, who have received chemoradiotherapy or radiotherapy, PD-1 inhibitor could significantly prolong the OS, and Pembrolizumab is likely to be superior to Nivolumab. Combination therapy with Pembrolizumab or Pembrolizumab monotherapy in first-line may prolong the OS. Moreover, based on NLR levels and sIL-8 levels, we may decide whether to choose immune checkpoint inhibitor as the treatment option and estimate the benefit from immunotherapy as well as the prognosis.

However, the above inferences mainly came from case reports or small sample studies and more animal experiments and clinical trial results are needed to help further confirm our insights.

In addition, the detection of targetable driver oncogenes could be attempted, and targeted therapy may be a potential treatment option. At the same time, we look forward to the clinical efficacy data of new targeted drugs, such as BETi, p300/CBP HAT inhibitor, HDACi, and dual HDAC/PI3K inhibitor.

Meanwhile, we proposed the possibility of anti-VEGF therapy combined with PD-1/PD-L1 inhibitor plus chemotherapy, PD-1 inhibitor combined with BETi, and IL-8 monoclonal antibody combined with immune checkpoint inhibitor to improve the prognosis in primary pulmonary NUT carcinoma.

## Author Contributions

XL and HS determined the writing direction and were responsible for the manuscript writing and modification. XL was responsible for literature collection and collation. WZ, CB, HH, and MH gave ideas and suggestions on selecting directions. MH and NT provided advice on pathology writing. YN, CF, and HQ took part in making charts. CB and YD provided financial support, and reviewed and revised the manuscript. All authors contributed to the article and approved the submitted version.

## Funding

This work was partially supported by the National Natural and Science Foundation of China(NO.82000102) and the Collaborative Innovation Cluster Foundation of Shanghai Health Commission(NO.2020CXJQ03).

## Conflict of Interest

The authors declare that the research was conducted in the absence of any commercial or financial relationships that could be construed as a potential conflict of interest.

## Publisher’s Note

All claims expressed in this article are solely those of the authors and do not necessarily represent those of their affiliated organizations, or those of the publisher, the editors and the reviewers. Any product that may be evaluated in this article, or claim that may be made by its manufacturer, is not guaranteed or endorsed by the publisher.
